# Extra Supplement—The Nursing Mirror

**Published:** 1889-01-26

**Authors:** 


[*Extra Supplement.*

### The Nursing Mirror.

All Contributions for this Supplement should be addressed "The Nursing Mirror," care of The Editor, 38, Old Jewry, E.C.

### En Passant.

UNACCUSTOMED DRUGS.—An American patient in a London Hospital asked the nurse for a drink; she handed him a glass of water. "Would you mind giving it me slowly in a spoon, nurse," said the patient, "till my system gets used to such a strange beverage."

DISTRICT NURSING IN EAST LONDON.—The East London District Nursing Society is going to undertake a new parish, that of Hoxton: this will make eight nurses under Miss Taylor. Aldgate badly needs a district nurse also, and, in point of fact, a fourth matron will soon be required if the society grows at this rate. The "Memoir of Alexander J. Ross, D.D.," just published, contains an interesting account of the formation and start of the L.N.S. under his supervision. This was in 1873.

SHORT ITEMS.—We regret to hear that Miss Annesley Kenealy has been seriously ill.—Two letters from nurses, begging Mr. Rathbone to join his fund to the National Pension Fund, appear in the *Liverpool Post*.—The nurses at Charing Cross are not allowed to talk during dinner.—The nurses of Liverpool Northern Hospital receive excellent lectures from the honorary medical staff.—The Chairman of the Nottingham Nursing Association appeals for funds to support the five district nurses now working for them.—The Guild tea at Barts. on Jan. 14th was a great success. Many nurses wore the Heartsease Badge.

COVENTRY NURSES.—The annual meeting of the Coventry District Nurses' Institution was held on January 10th, Lord Leigh presiding. Two sisters, the Misses Wing, do their best to nurse the sick poor in a population of 50,000. Last year 247 cases were registered on the books, and an average of 24 visits a day were paid. Finances are satisfactory; so perhaps a third nurse may be started this year. There is an objectionable plan by which the workhouse at Coventry sends out inmates called nurses, who get half-a-crown a-week. The institution should make this unnecessary.

NURSE AND STEWARDESS.—Would it serve a useful purpose to open a register for nurses who would like to go on voyages as stewardesses? There are several shipping companies who appreciate the idea, but we have not yet heard of any nurse who is willing to be the pioneer of this movement. Certainly nurses ought to sail in the North Sea Fisheries Hospital Ships, two at a time, in the same way that surgeons now go on a cruise in these boats, and combine business and pleasure. There is always the question of sea-sickness to be faced: it is such a pity the rose has a thorn!

NIGHT NURSING.—The Workhouse Infirmary Nursing Association has issued a pamphlet on the subject of Night Nursing in Hospitals and Infirmaries. The opinions and suggestions of Miss Twining, Miss Wood, Miss Mollett, Miss Hanan (of Crumpsall), and Dr. Benton (of the Central London Sick Asylum) are all quoted, and form a striking protest against the almost universal infirmary system of permanent night nurses. All these authorities agree that (1) long periods of night work are injurious to health, and to a high standard of work. (2) That the most satisfactory system of night nursing is to appoint assistant nurses, or fully trained probationers, on alternate day and night duty for three or four months at a time. (3) That the general health and comfort of night nurses should be specially considered, as their work is so trying. (4) That charge nurses should never be changed to night work. There can be no doubt as to the justness of the above conclusions, and we hope they will be well considered by all authorities connected with nursing establishments not formed on these lines.

IRELAND WAKING UP.—We should scarcely expect to find advanced views of nursing amongst the Guardians of Coleraine, but these gentlemen are wiser than many of our English Boards. The late nurse of Coleraine Infirmary had to be summarily dismissed, whereupon the ratepayers petitioned the Board to give them a respectable and efficient nurse, and to re-arrange the infirmary on a more orderly and decent system. The Medical Officer approved of this memorial. The Board thereupon proceeded to advertise for a trained nurse to fill the vacant appointment, at a salary of £25. They also undertook to consider the need of nursing and surgical appliances at the next meeting.

SCIENTIFIC LECTURES.—The amateur nurse who does nearly all the writing about nursing is beginning to take to lecturing on this subject as well. The St. John's Ambulance Association started the craze for nursing lectures, and several women have been clever enough to turn it to their own account, and with little knowledge, but lots of cheek, go touring in the provinces to deliver lectures on Nursing. Dr. Lauder Brunton tells an amusing story about such a lecture: "The lecturer described the different methods of preparing gruel, and observed that whenever the gruel was required for a case of bronchitis, a piece of butter should always be added to it, 'because,' said she, rubbing her chest with her hand, 'butter is so very healing to the inside.' She was evidently under the impression that the piece of butter got into the chest, ran all about, and thoroughly greased the air-passages."

DISTRICT NURSING.—The Paddington and Marylebone District Nursing Association has sent out a statement for the consideration of the richer dwellers within its radius. At the end of 1888 the Association finds itself in debt to the extent of £150, and unless means can be procured to meet this deficit the committee will be obliged to reduce their expenses by withdrawing the nurse set apart for infectious cases. This would be a great misfortune to the poor resident in the surrounding parishes. At the annual meeting last June it was hoped rather to increase the staff by adding a sixth nurse, for whom there is plenty of work in the district and a room in the home. The number of patients nursed last year, including infectious cases, exceeds 900, and by far the greater part were women and children. The total number nursed during the whole of 1887 was 923. The committee do not employ a collector, and subscriptions should be sent to the Lady Superintendent, Miss Perssé, at the Home, 510, Edgware-road, W.

ARMY NURSES.—The *Tablet* of January 5th has a long article by an army nurse, giving details and anecdotes of her life and work, the special point insisted on being the unsectarianism of military hospitals. The writer trained at the Middlesex, where she found her religion in no way a disadvantage; she then went to Netley, thence to Chatham, and then to Portsmouth, as Superintending Sister. At Netley there is a chapel which is used every Sunday for services conducted by a Catholic priest, a Church of England chaplain, a Wesleyan and a Presbyterian minister, so that both patients and nurses have their choice of religions. The army nurse's headquarters are Netley, but Woolwich, Portsmouth, Chatham, Aldershot, Dover, Shorncliffe, Devonport, Gosport, the Guards in London, Canterbury, one or two hospitals in Ireland, besides Gibraltar, Malta, and Cairo, all possess a staff of nursing sisters. The writer tells a touching anecdote of the Queen's visit to her wounded soldiers brought back from Egypt. One lad she spoke to had had his leg amputated, and though he simply answered "Yes, Majesty," and "No, Majesty," to the kind questions put to him, the excitement was too much, and that night hæmorrhage set in. Her Majesty, hearing of this, used to telegraph to inquire after the young fellow, and expressed her pleasure when he finally recovered.

### Original Articles.

### THE NATIONAL PENSION FUND.

"Blessed is the man who knoweth his own mind," and I am of opinion it is certainly time everyone engaged in the profession of nursing should know their own mind with regard to the Pension Fund. I am quite sure that whatever differences of opinion may exist as to the benefits likely to accrue to nurses through it, there can be but one view held as regards the kind thoughtfulness which started it. In writing the present article, I wish it to be distinctly understood I am not writing against the fund, nor yet am I entirely in favour of it.

I would first speak of the shilling a-month table, advocated chiefly because it is bound to meet the resources of every nurse. I am at a loss to understand what object a nurse could have in view in laying aside so small a sum of money. Just suppose that a nurse at the age of fifty or sixty is completely disabled from work, is wholly dependent on the product of a shilling a-month, which twenty-five years ago she commenced to lay aside for the evil day. What a grim reality it would prove to a nurse to exist at sixty on £2 14s. 2d. per annum! To talk about small pensions being supplemented by bonuses, etc., is, of course, some comfort, but is it wise on joining a fund of this sort to count on more than one is justly entitled to claim? It may be asked, Then why do not nurses lay aside a larger premium for a sufficient pension? Simply because till they are out of their three years of probation they could not possibly afford to do so. It must not be overlooked that, with few exceptions, in London women do not begin to train till the age of twenty-five, and that their salaries range from £8 to £14 a-year for the first year, and not over £20 for the third year; consequently they are twenty eight years of age before they can hope to obtain a reasonable salary. At twenty-eight the nurse, to gain a pension of £15 per annum at the age of fifty, must lay aside, according to Table A, 12s. 6d. per month; or, on the returnable Table B, 14s. 3d. per month, and it requires but a trifling calculation to see how large a proportion of her earnings must go in premiums to this fund.

I must proceed to another objection, or fancied objection. There has been some discontent manifested on the score that nurses have to pay more for their pensions than the men. Most people know that all insurance companies and deferred life annuities are conducted on the principle that the average duration of a woman's life exceeds that of a man's, and it is unreasonable to expect the National Pension Fund to do what has been found impossible in other companies. But is there to be no difference between a public insurance office and one specially meant for nurses? I think this question could only be answered by ascertaining the average duration of a nurse's life. Is it the same as any other woman's? I am under the impression that it is not, that nurses die "used up" sooner than most women. I have read some statistics about this, but I cannot at this moment recollect where I saw them. If this is true, could not the rule of higher premiums for women be modified in a special fund for nurses? We should like a little more information on this point.

If at the expiration of two years a nurse, having previously entered under Table B, desires to withdraw her contributions, does she not receive it back intact, but without even the small interest offered to those who place their money in the Post Office Savings Bank?

Do those who urge us to lay aside for a bare existence at fifty or sixty years of age realise what a life of continuous self-denial it entails upon us? Some time ago The Hospital suggested we should make better use of our opportunities to go to some of the cheap concerts. Laying aside the fact that the hours when nurses are off duty and the hours when concerts are on are for the most part widely different, it is easy to see that all such amusements could not form part of the recreation of nurses striving to save. All the small enjoyments which go to make up the happiness of life must be rigorously excluded. Unless free tickets can be obtained, exhibitions, concerts, art galleries, etc., all must be closed to the "saving" nurse; and last, but not least, the little presents here and there, and all the small comforts nurses like to collect around them.

We know well enough men practise stern self-denial in their early days, but not for an end such as ours would be, not for a bare existence in old age. The difference to me is very marked; whilst man toils with the hope of comfortable independence in the future, the nurse works equally hard in her way, with equally long, if not longer, hours, just to keep body and soul together now and in the future (a future which may never come)—and for such a future! Of course, the whole argument is based on the "if;" *if* we do not marry, *if* we do not change our profession, *if* we do not fall out of the nursing ranks, *if* we should happen to live to be fifty or sixty. We hear vague hints from authorities that "they could a tale unfold" as to the miseries and hardships endured by nurses when no longer able to work. I know personally of six nurses who died last year, all young women, however; but one can scarcely call death a fate, nor would they perhaps consider their premature end was a calamity. I did hear of one case which, to be fair, I ought to mention. A lady told me that hearing her old nurse was ill, she went to see her, and found her dying at the age of fifty-five of that peculiar, but alas! common disease, "used up;" the neighbours called it "a waste," but this lady told me nurse did not seem to be suffering from any special illness. Her death was rather sad: she was alone, and dependent on the tender mercies of the neighbours. On a chair by her bed was a cup of weak tea. which every now and then she raised to her parched lips. The end was near, the mind—a once strong mind—was wandering; in her delirious mutterings, one could distinguish a broken word or so, showing how her troubled thoughts still ran on pulses, temperatures, doctors. All through the long night the poor body lay tossing about in restless anguish, till with the break of dawn, the shadows passed away, the weary eyes closed, one more prolonged agonizing shiver—the nurse rested.

Yet another thought occurs to me. What is to be done with those nurses who are already ill, who have developed various diseases which prohibit the hope of a long life, and who at an early age will be thrown helpless on the hands of relatives not too willing to bear the burden thus cast upon them? I have known of nurses suffering from heart disease, as a sequela of rheumatic fever; of others with chronic swellings of the legs and ankles; and, again, of others who have developed epilepsy. Who is to help these, and what can be done to relieve their pressing necessities? How much better for our self-respect if only our salaries could be raised, and our work be properly remunerated; then we should be able to think more hopefully of our future, and without all the strain and self-denials it now entails upon us.

I must conclude, not without a prayerful hope that, whatever befals us individually, our fate may not be as the fate of Mrs. Sarah Duyck, nor our nursing-end such as hers.

What a warning to us all not to apply for the "proposed method of assistance" as originated by "A Contemporary." I have read the name for months, and positively my dreams are disturbed by apparitions of Mrs. Duyck in an unhallowed luminous glow of votes, and her broken arm raised menacingly above me. Handclasp.

[Handclasp's questions are answered in the next column.—Ed. *T. H.*]

### THE REPLY OF THE PENSION FUND AUTHORITIES.

MR. EDWARD T. CLIFFORD, the manager of the Pension Fund, has kindly sent us the following reply to the points raised by "Handclasp" in the foregoing letter :—

By your courtesy I have been favoured with a proof copy of the article signed "Handclasp," to be inserted in this week's issue of THE HOSPITAL, on the National Pension Fund for Nurses, and am pleased to have the opportunity of supplying answers dealing with the criticisms and inquiries contained therein. It is probably beyond my competence to treat adequately some of the points in the article, inasmuch as they refer to nursing questions outside the limit of my experience as an assurance secretary. But some of the points raised are of prime importance to nurses, and must be considered by the Pension Fund in so far as they come within the range of its action. Perhaps no stronger argument in favour of the National Pension Fund was ever penned by any critic, favourable or adverse, than "Handclasp's" article. It does not touch the question of the value of this particular Fund, but it demonstrates to absolute conviction the need for a fund of some kind, and especially for one which shall be liberally helped by a sympathising and generous public. This is the central question : Seeing that some kind of fund is indispensable, is it better to have a purely charitable fund, or one which shows a disposition on the part of nurses to help themselves so far as is possible? The answer cannot be for a moment doubtful. It is as certain as anything can be that the public will give infinitely more to a fund which shows self-dependence on the part of nurses than they would to a purely charitable movement; while nurses themselves, if they are like ordinary mortals, cannot but feel that when they have done all that is possible with their limited means to provide for their own future, they have amply saved their self-respect. The subject of rates it would be futile to discuss again for the hundredth time. It may be said, once for all, that it is one with which actuaries alone can deal; and it is sufficient for nurses to know that if the pensions do not cost the contributions charged, the balance will be returned to them as profits. As Dr. Potter pointed out at St. Thomas's Hospital on the occasion of the reading of my paper there, the Fund is the nurses' own; and it is for them so to use it and avail themselves of it so as to bring into sympathy with themselves and it the whole of that generous public whom they so self-denyingly serve. I consider that this answer on the subject of rates deals sufficiently and convincingly with many of the side issues raised in the article of "Handclasp" as well as with the rate question. But there are certain other difficulties which it hardly touches, and with these I will deal a little more in detail. First, as to the 1s. a month tables; these were inserted simply and solely by way of illustration, and with the object of showing nurses the purchasing power of one shilling a month. But it was never contemplated that nurses who have a weekly income of eight or nine shillings would be content to take out such a pension as one shilling a month would produce. It was only intended to show that if 1s. a month would produce so much, 2s. would produce double, and 4s. four times that amount and so on. It was not our purpose to ask nurses to enter for such a pension as 1s. a month would provide, but so to simplify the actuarial facts of the case as to make them see at a glance what they would or would not be able to do. With regard to probationers, it is not to be expected that whilst they remain probationers they can enter for a very large pension. But they can, even as probationers, at least join the Fund with what they are able to afford at the moment, if only on the shilling a month scale; and having done so, they can with advantage to themselves rise to an increased pension when their increased salaries enable them to take that step. The point to be borne in mind is that the earlier they become members of the fund, even at the lowest of all scales of contributions, the smaller will be the annual payments and the total amount they will give for their pensions. Surely after their probationary period is over, and they are in receipt of a salary of, say, £20 to £30, they can, if so disposed, contribute such a monthly sum as will give them a pension well worth having, and which the Council of the Fund are determined, with bonuses, shall be sufficient to meet their real necessities and to raise them above want. In dealing with the next objection, viz., that the rates for females are higher than those for males, it is sufficient to point out that, especially on the returnable scale, the difference is slight, and that both sets of rates have been calculated on precisely similar principles, *i.e.*, upon scientifically constructed mortality tables. Whatever difference there is arises solely from the ascertained difference between male and female longevity. With regard to the idea that female nurses are less-long lived than male nurses, I would point out that there are no facts to justify such an assumption, but that on the contrary the probabilities are all the other way. We are dealing with nurses in both cases. Moreover, it should be remembered that male nurses do not participate in the bonuses from donations, but simply in the profits created by their own policies. It is, perhaps, worthy of remark that the only other Nurses' Pension Fund now in existence shows that, as a matter of actual experience, the average age of its present pensioners is found to be 76 years. With regard to the inquiry as to returnable contributions, the same principles are adopted by the Fund as are in force in the Government Annuity Office. No interest is at present paid when contributions are returned, but they will probably be returnable later on with the addition of certain bonuses to be declared from time to time at the Council's discretion. If a nurse should at any time find her contributions more than she can afford, the Council are prepared to meet her case by recasting the pension. No comparison can be made between those contributing to this Fund and those depositing in the Post Office Savings Bank; because, while the money remains with the Fund, it is qualifying for a much larger result, by way of pension, than a similar sum deposited at interest in the Savings Bank. Moreover, contributions to the Fund are simultaneously qualifying for a share in the profits and bonuses, as these will be allotted in proportion to the premiums. Finally, the sick pay tables will obviate the necessity for any future distress such as "Handclasp" records. It is clear, therefore, that from every point of view a nurse will do better for herself by joining the National Pension Fund than she can possibly do anywhere else.

### A Christmas Holiday.

*(Continued from page lix.)*

### GUILDFORD AND ITS HOSPITAL.
### PART II.

WE arrived at Guldford that Saturday night about six o'clock, and wandered down the picturesque High Street, not too tired to look into the windows of the curiosity shops. Guildford is a city which is builded upon a hill, and is thoroughly quaint and interesting. There are no two houses alike in its straggling streets, and gables and windows of fantastic form, predominate largely. It was market night, and the town was very busy; but we gradually made our way down the steep hill, across the bridge, into the quieter quarters where my friends dwelt. They were not in but were expected shortly, and we were offered a cup of tea in the meantime. Of course we accepted, and then we strolled to the window, and looked out. To our right rose the town, with its innumerable lights all burning; to our left lay great masses of rolling downs, dark against a faint sky, in which hung the pale sickle of a new moon. Just opposite the windows was a large building, brightly lit.

"What place is that?" asked Nurse Sadler.

"The Surrey County Hospital; didn't you guess it was?" I said. "Let's go across and see it, and so pass the time till these people return."

"What! after walking nearly thirty miles?"

"Oh, nonsense! Say twenty. But do come."

So we went across to the hospital, and asked for the matron, but of course she was too busy to attend to us, and handed us over to a head nurse. There are no "sisters" at Guildford; the committee don't like the sound of the word, though it is such a pretty and convenient title. Two large

Christmas trees stood in the hall, waiting to be decorated and taken upstairs—one for the children and one for the adults. The old wards of the Surrey County I knew well, with their nice dark floors, and great windows opening into a balcony, where you can wheel the patients on a fine day. And what a view there is from that balcony! Certainly no other hospital has a more picturesque situation to my mind than the Surrey County. The wards looked specially nice because they were all decorated with masses of evergreens and cheerful texts. The patients looked happy and expectant, and all boded well for a merry Christmas. I noticed a good many improvements, including less crowding, though the in-patients now number about five hundred a-year. This is of course the result of the new wing, which was really the one thing that drew me across to the hospital at that late hour. Our guide kindly led us towards the new building, and we entered the children's ward, large and light. The walls were not yet colour washed, and there was an incomplete air about the big ward:—

"But that time will remedy," said Nurse; "and now we are going to cover up all deficiences with holly."

"But I thought the new wing was to hold two large wards, as well as smaller ones?"

"Yes, there is the other ward," said Nurse, "throwing open a door into a pitch dark place; "it is unused for want of funds. The old story."

"What a pity! But I'm sure it won't be so long. You always were, and always will be, well taken care of at the Surrey County. How do you manage about these small wards; who looks after them?"

"Oh, if I have a patient or patients here, I have to run round; and it is rather inconvenient being so far from the other wards. I have a man here with brain mischief just now; that's why these rugs are down to deaden the sound. I mustn't take you in, as the slightest noise hurts."

We said good-bye to our guide, and departed from this cheerful hospital, and made our way back to my friends. They had returned, and were waiting to make much of us after our long tramp. We could not keep awake after dinner, and at nine o'clock we ignominiously said good night, and went off to bed and slept like tops till the following morning.

(*To be continued.*)

### Lectures.

An esteemed correspondent writes:—On January 18th Mr. Brudenell Carter delivered an excellent lecture on "The Scientific Aspects of Truthfulness" at the Medical Societies' Rooms in Chandos Street. There were about fifty nurses present, and one gentleman. Dr. Buzzard, of the National Hospital, took the chair. Mr. Brudenell Carter commenced by drawing an illustration from the barometer, and the different theories which account for its rise and fall. Coming nearer nursing matters, he spoke of the clinical thermometer, from the date of the introduction of which he based the commencement of scientific nursing. The use of slipshod language and of inaccurate phrases was strongly condemned by Mr. Brudenell Carter, who bade all nurses carefully separate their facts from their opinions. As an illustration of his meaning, the lecturer imagined a case of a rise in a patient's temperature after a meal of solid food. Here the fact is the rise of temperature. The opinion is, that it was caused by the food. Scientific truthfulness would lead the nurse to state, "The patient's temperature rose so many degrees last night, I think owing to a meal of solid food." She would not omit the "I think." Women are only too ready to fall into a careless manner of talking, which is especially reprehensible in nurses, and Mr. Carter's plea for greater accuracy and more scientific truthfulness should be heeded by all. Speaking of the exaggeration and want of exactness, not to say wilful perversion of facts, which is found in a certain class of newspapers, the lecturer waxed quite eloquent and indignant. This was rather rough on the number of contributors to one of your contemporaries who were present, and who could scarcely have expected their style of journalism to be condemned by their pet association. A discussion followed the paper, during which Miss Mollett made some amusing and interesting remarks, stating that for her part she thought life would be intolerably dull if deprived of fiction. We think Miss Mollett must have been reading Oscar Wilde's article in the *Nineteenth Century* on the "Decay of Lying." Miss Wood and Miss Homersham spoke, dragging in the question of "revealed truths," though Mr. Brudenell Carter had, with the best taste, carefully avoided touching on religious questions. Sister Henrietta, of University College, Dr. Bedford Fenwick, and others also spoke.

### Appointments.

[It is requested that successful candidates will send a copy of their applications and testimonials, with date of election, to The Editor, The Lodge, Porchester Square, W.]

Watford Cottage Hospital.—Miss Hillyer has been appointed matron of this infirmary. Miss Hillyer trained at Middlesex, and for some time acted as sister-superintendent of the Nursing Institute connected with that hospital.

British Nurses Association.—It is announced that one of the honorary secretaries, Miss Catherine J. Wood, has been appointed secretary to this association. The salary and emoluments of the office are not stated.

Ulverston and District Cottage Hospital.—Mrs. Cox, late matron of the Metropolitan Hospital, Kingsland Road, has been appointed matron of this hospital.

### Vacancies.

The following vacancies are announced:—

**Matrons.**

Lying-in Hospital, Brownlow Hill, Liverpool.
Cromer Cottage Hospital.
Matlock Convalescent Home; £40.

**Assistant Matron.**

Lying-in Hospital, Brownlow Hill, Liverpool.

**Nurses.**

Nursing Institution, 96, 97, 98a, Wimpole Street, London, W., Mr. Wilson has several vacancies for thoroughly-trained Nurses.

Royal Albert Hospital, Devonport; £25.

Cardiff Infirmary.

Croydon General Hospital (Night); £22.

Southport Infirmary; £20.

Llandudno Cottage Hospital.

Workhouse Infirmary Nursing Association.

Blackheath Institute; £20.

Swansea Nursing Institute.

Nursing Institute, Bangor, North Wales, several; £20.

Rivers Street Home, Bath (several); £25.

Nurses' Institute, Canterbury (four).

Truro Trained Nurses' Home; £25.

Nurses' Home, Ellison Place, Newcastle.

Southport Nurses' Home (several).

Nurses' Home, St. Heliers; £25.

Nursing Home, Brockley.

Nurses' Home, Norwich.

County Institution, Worcester.

Nursing Institute, Ryde.

Newport Infirmary; £25.

Wonford House, Exeter.

St. Bede's House, Fairfax Road, N.W.

**District Nurses.**

Newlands, Ryde.
Bedford Nursing Institute.

**Probationers.**

Bolton Infirmary,

Workhouse Infirmary Nursing Association.

Ellison Place Home, Newcastle.

Tewkesbury Hospital.

Great Yarmouth Hospital.

**Wardmaid.**

Royal Hospital for Diseases of the Chest, City Road.

**Masseuse.**

Nursing Institute, Ryde.

### Notes and Queries.

To Correspondents.—1. Questions may be written on post-cards. 2. Advertisements in disguise are inadmissible. 3. In answering a query please quote the number. 4. If a private answer is desired, a stamped addressed envelope must be forwarded.

**Queries.**

(102) *Training in Midwifery.*—Can anyone tell me where a nurse in her fourth year could get free training in midwifery in return for her services?—*T.M.*

(104) *Starting a Crèche.*—Information wanted as to how to start and manage a crèche.—S. P.

**Answers.**

(88) *Health Lectures.*—The Edinburgh Health Lectures are published by Messrs. Macniven and Wallace, of Princes Street, Edinburgh.

(94) *Nurse James.*—Try the north coast of Devon.

(97) *Home Wanted.*—We have received too many replies to this query to print them. They have all been forwarded to *Nurse J.*

(101) *Nursing in France.*—Apply to the Superintendant, Nouvelle Hôpital, Rue de Condé, Havre.

(99) *Brighton.*—Do not go as a paying probationer. Consult the *Hospital Annual* for list of hospitals, and apply to the matron of any you fancy for particulars. Choose one, if possible, without a private nursing branch.—*A. M. T.*

(103) *Somerville Club.*—"Temperance" is informed that the Somerville Club is at 231, Oxford Street, W., and the secretary will supply particulars.

